# Gangliosides in the Brain: Physiology, Pathophysiology and Therapeutic Applications

**DOI:** 10.3389/fnins.2020.572965

**Published:** 2020-10-06

**Authors:** Simonetta Sipione, John Monyror, Danny Galleguillos, Noam Steinberg, Vaibhavi Kadam

**Affiliations:** Department of Pharmacology, Faculty of Medicine and Dentistry, Neuroscience and Mental Health Institute, University of Alberta, Edmonton, AB, Canada

**Keywords:** gangliosides, Huntington’s and Parkinson’s diseases, Alzheimer’s disease, hereditary spastic paraplegia, GM1, epilepsy, depression, neurodegeneration

## Abstract

Gangliosides are glycosphingolipids highly abundant in the nervous system, and carry most of the sialic acid residues in the brain. Gangliosides are enriched in cell membrane microdomains (“lipid rafts”) and play important roles in the modulation of membrane proteins and ion channels, in cell signaling and in the communication among cells. The importance of gangliosides in the brain is highlighted by the fact that loss of function mutations in ganglioside biosynthetic enzymes result in severe neurodegenerative disorders, often characterized by very early or childhood onset. In addition, changes in the ganglioside profile (i.e., in the relative abundance of specific gangliosides) were reported in healthy aging and in common neurological conditions, including Huntington’s disease (HD), Alzheimer’s disease (AD), Parkinson’s disease (PD), amyotrophic lateral sclerosis (ALS), stroke, multiple sclerosis and epilepsy. At least in HD, PD and in some forms of epilepsy, experimental evidence strongly suggests a potential role of gangliosides in disease pathogenesis and potential treatment. In this review, we will summarize ganglioside functions that are crucial to maintain brain health, we will review changes in ganglioside levels that occur in major neurological conditions and we will discuss their contribution to cellular dysfunctions and disease pathogenesis. Finally, we will review evidence of the beneficial roles exerted by gangliosides, GM1 in particular, in disease models and in clinical trials.

## Introduction

**FIGURE 1:**
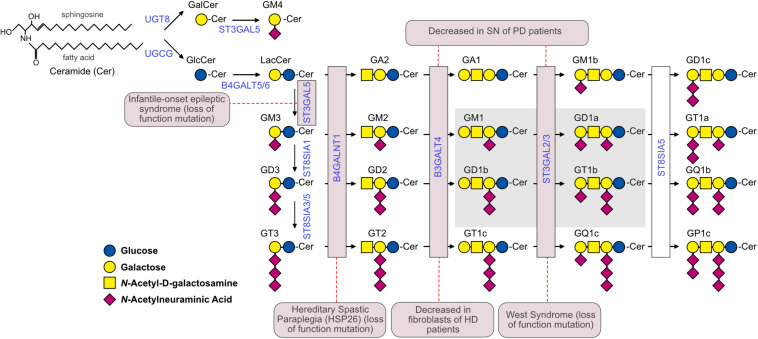
The ganglioside biosynthetic pathway and related pathologies. Biosynthetic enzymes are indicated in blue and are boxed in pink if mutations or altered expression are associated with specific neuropathological conditions. The Svennerholm’s nomenclature (Svennerholm, 1994) was used to identify gangliosides. The G at the beginning of each ganglioside name indicates the belonging to the ganglio-series of glycosphingolipids; A, M, D and T indicate the presence of zero (absent), one (mono-), two (di-) and three (tri-) sialic acid residues, respectively. All gangliosides with the nuclear core structure Galβ1-3GalNAcβ1-4Galβ1-4Glcβ1-1’Cer are indicated with the number 1, gangliosides lacking the terminal galactose are indicated with the number 2, and gangliosides lacking the terminal disaccharide galactosyl-*N*-acetylgalactosamine (Galβ1-3GalNAc) are indicated with the number 3. GM4 is a ganglioside of the gala-series. The 0, a-, b-, and c-series include gangliosides with 0, 1, 2, and 3 sialic acid residues linked to the innermost galactose, respectively. The simple gangliosides GM3, GD3, and GT3 serve as the precursors for the complex gangliosides of the a-, b-, and c-series. The most abundant brain ganglioside species are boxed in gray. The dashed red lines connect enzymes with the corresponding pathology. In the case of West syndrome, the disease has been associated to a mutation in ST3GAL3, while in the case of PD, ST3GAL2 expression was shown to be affected. UGCG, UDP-Glucose Ceramide Glucosyltransferase; UGT8, UDP-Galactose-Ceramide Galactosyltransferase; B4GALT5/6, UDP-Gal:Beta-GlcNAc Beta-1,4-Galactosyltransferases 5 and 6; ST3GAL5, CMPNeuAc:Lactosylceramide Alpha-2,3-Sialyltransferase; ST8SIA1, ST8 Alpha-*N*-Acetylneuraminide Alpha-2,8-Sialyltransferase 1; ST8SIA3/5, ST8 Alpha-*N*-Acetyl-Neuraminide Alpha-2,8-Sialyltransferases 3 and 5; B4GALNT1, UDP-Gal:BetaGlcNAc Beta-1,4 *N*-Acetylgalactosaminyltransferase 1; B3GALT4, UDP-Gal:BetaGlcNAc Beta 1,3-Galactosyltransferase 4; ST3GAL2/3, CMP-*N*-Acetylneuraminate-Beta-Galactosamide-Alpha-2,3-Sialyltransferase 2; ST8SIA5, ST8 Alpha-*N*-Acetyl-Neuraminide Alpha-2,8-Sialyltransferase 5.

Gangliosides are glycosphingolipids composed of a ceramide lipid tail attached through glycosidic linkage to a glycan headgroup containing one or more sialic acid residues (Figure 1). In the nervous system, gangliosides are the main carriers for sialic acid (Schnaar, 2004), a terminal sugar that decorates the surface of cells and plays important roles in cell-cell and pathogen-cell interactions (Varki et al., 2015). Gangliosides are most abundant at the plasma membrane (van Meer and de Kroon, 2011). Their hydrophobic ceramide tail inserts into the outer phospholipid leaflet, while the glycan headgroup extends outwardly and engages in *cis* (within the same membrane) as well as *trans* interactions (with molecules on other cells and in the extracellular space) that result in modulation of cell signaling and cell-to-cell communication (Posse de Chaves and Sipione, 2010).

Gangliosides are synthesized in the Golgi apparatus from which they are mainly trafficked to the plasma membrane (van Echten-Deckert and Gurgui, 2008; Yamaji and Hanada, 2015). Due to intracellular membrane trafficking, gangliosides can also be found in the endo-lysosomal system (Möbius et al., 1999; Chinnapen et al., 2012), at endoplasmic reticulum (ER)-mitochondria contact sites (Sano et al., 2009; Sorice et al., 2012) and even at the nuclear envelope (Xie et al., 2002; Tsai et al., 2016).

The heterogeneity in the sugar composition of the glycan headgroup gives rise to >200 ganglioside structures. Additional molecular diversity is conferred by the ceramide moieties, which include long-chain bases (sphingosine) and fatty acyl chains of different lengths and various degrees of saturation (Sonnino and Chigorno, 2000; Yu et al., 2011). In spite of these remarkable variety of structures, the bulk of gangliosides in Vertebrates is made up of only a few major ganglioside classes (Figure 1). For a detailed discussion on ganglioside nomenclature and biochemistry, the reader is referred to other reviews on this topic (Kolter, 2012; Schnaar, 2015; Kopitz, 2017).

**FIGURE 2:**
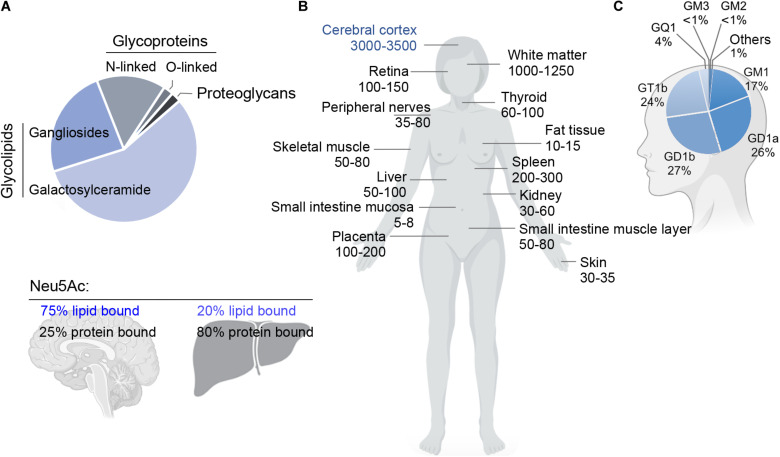
**(A)** Relative abundance of glycoproteins, glycolipids and proteoglycans in the adult rat brain (top), and fraction of sialic acid (Neu5Ac) bound to lipids (gangliosides) or proteins in brain and liver (bottom). Glycosphingolipids represent ∼80% of the total mass of glycans and carry ∼75% of the sialic acid residues in the adult brain. Data adapted from Schnaar (2004) and Schnaar et al. (2014). **(B)** Concentration of gangliosides in human organs (nmol NeuAc/g of fresh tissue weight) (left). Data adapted from Svennerholm (1980). **(C)** Relative abundance of individual gangliosides in the human brain (right). The human brain expresses a higher proportion of gangliosides of higher complexity compared to other tissues. Data adapted from Tettamanti et al. (1973).

Total mass and specific ganglioside composition vary significantly from tissue to tissue and among different cell types. The human brain contains 10- to 30-fold more gangliosides than any other tissue or organ in the body (Figure 2; Svennerholm, 1980). Simple gangliosides, like GM3, predominate in most peripheral tissues (Prokazova et al., 2009), while >90% of the brain ganglioside mass is constituted by four complex gangliosides (GM1, GD1a, GD1b, and GT1b) (Figure 1; Tettamanti et al., 1973). The relative abundance of specific gangliosides in different brain areas and cell types has not been investigated in a systematic and comprehensive manner yet. However, early studies suggested that gangliosides are most abundant in myelin, followed by neuronal cells (Yu and Iqbal, 1979). Along with GM1, myelin also contains high amounts of GM4 (Figure 1; Yu and Iqbal, 1979), which is also found, together with GM1 and GD3, in adult oligodendrocytes (Kim, 1990). In contrast to neurons, astrocytes mainly produce simple gangliosides, including GM3, GD3 and, to a lesser extent, GM4 (Asou and Brunngraber, 1983; Sbaschnig-Agler et al., 1988; Ozawa et al., 1993). Little is known about gangliosides in microglia, except that they display GM1 and, when activated by pro-inflammatory stimuli, significant amounts of GD3 (Nedelkoska and Benjamins, 1998; Simon et al., 2002).

Remarkably, gangliosides account for 80% of all glycans and >75% of the sialic acid present in the brain. This is in stark contrast with most other organs and tissues, where >80% of glycans and sialic acid residues are linked to glycoproteins (Figure 2; Schnaar, 2004). Since glycans and sialic acid are crucial players in the communication between cells (Gabius, 2009), their striking over-representation in brain gangliosides explains the pivotal role that the latter have in cell signaling and cell-cell interactions in the nervous system.

Gangliosides are essential for the maturation and maintenance of the central nervous system (CNS). Loss-of-function mutations in genes that encode ganglioside biosynthetic enzymes result in severe neurodevelopmental and neurodegenerative disorders in humans (Simpson et al., 2004; Boukhris et al., 2013; Fragaki et al., 2013; Harlalka et al., 2013; Boccuto et al., 2014; Wakil et al., 2014) and in mouse models (Allende and Proia, 2014). A decrease in ganglioside levels and changes in the relative abundance of specific gangliosides might also occur in aging (Segler-Stahl et al., 1983; Palestini et al., 1990; Kracun et al., 1992b; Mo et al., 2005) and in common neurodegenerative conditions, including Alzheimer’s disease (AD), Parkinson’s disease (PD) and Huntington’s disease (HD) (Blennow et al., 1991, 1992; Desplats et al., 2007; Maglione et al., 2010; Wu et al., 2012), among others (Figure 1 and Table 1). This raises two main questions: Does a decline in the synthesis of gangliosides contribute to the onset and/or progression of common neurodegenerative conditions? Could interventions that restore normal ganglioside levels be neuroprotective? In this review, we will summarize ganglioside functions that are crucial to maintain brain health, we will review changes in ganglioside levels that occur in major neurological and neurodegenerative conditions and will discuss how these changes might contribute to cellular dysfunction and disease pathogenesis. Finally, we will review evidence of the neuroprotective role exerted by exogenously administered gangliosides, GM1 in particular, in disease models and in clinical trials.

**TABLE 1:** Changes in gangliosides during aging and across different neuropathological conditions.

Condition	Species	Stage, Type or Model	Gangliosides	Sample	Method	References
Aging	human	>90 yo	↓Gangliosides	FCx, TCx	HPTLC-Resor.	Svennerholm et al., 1991, Svennerholm et al., 1994
		20–90 yo	↓GM1, GD1a (a-series)	FCx	TLC-Resor.	Kracun et al., 1991
		20–90 yo	↓GT1b, GD1b (b-series)	Cb Cx	TLC-Resor.	Kracun et al., 1991
		20–90 yo	No changes	OCx, Hip	TLC-Resor.	Kracun et al., 1991
HSP	human		↑GM3; ↓GM2	Fibroblasts	HPLC, MS	Harlalka et al., 2013
S and P	human		Lack of GM3	Fibroblasts	TLC-Orcinol, MS	Boccuto et al., 2014
Epilepsy	human	Infant, West syndrome	↓GM1, GD1a	CSF	TLC-immuno	Izumi et al., 1993
		Infantile-onset symptomatic epilepsy	Lack of GM3 and derivatives	Plasma	HPLC	Simpson et al., 2004
	rat	Pilocarpine epilepsy	↓GM1, GD1a, GD1b, GT1b	Hip	TLC-Resor.	de Freitas et al., 2010
	mouse	Amygdaloid-kindled seizures	↓GM1; ↑GQ1b	Hip	HPTLC	Kato et al., 2008
PD	human	↑ ave. 16y of disease	↓GD1a, GD1b, GT1b	SN	HPTLC	Seyfried et al., 2018
		ave. 13.5y of disease	↓GM1, GD1b, GT1b	SN	NP-HPLC	Huebecker et al., 2019
		ave. 15.3y of disease	↓GM1, GD1a, GD1b	SN	NP-HPLC	Huebecker et al., 2019
		ave. 17.4y of disease	↓GD1b, GT1b	SN	NP-HPLC	Huebecker et al., 2019
		H and Y score 1(6), 2(21), 3(3)	↑GM3; ↓GM2, GD3, GD1a, GD1b, GT1b	CSF	NP-HPLC	Huebecker et al., 2019
		H and Y score 1(6), 2(21), 3(3)	↓GM1, GD1a	Serum	NP-HPLC	Huebecker et al., 2019
HD	human	Vonsattel 3	↑GD3	Caudate nucleus	HPTLC	Desplats et al., 2007
		Vonsattel 3	↑GM1	Cb	HPTLC	Denny et al., 2010
		Fibroblasts	↓GM1		ChTxB	Maglione et al., 2010
	mouse	Primary neurons	↓GM1		TLC	Maglione et al., 2010
		YAC128 5mo	↓GD1a, GT1b	CC	ChTxB, Immunoblot	Di Pardo et al., 2016
		YAC128 6mo	↓GM1, GT1b	Cx	TLC, ChTxB, Immunoblot	Maglione et al., 2010; Di Pardo et al., 2016
		YAC128 6mo	↓GM1, GD1a	St	ChTxB, Immunoblot	Maglione et al., 2010; Di Pardo et al., 2016
		YAC 128 9mo	↓GM1, GD1a, GT1b	CC	ChTxB, Immunoblot	Di Pardo et al., 2016
		R6/1	↓GM1	FB	HPTLC	Desplats et al., 2007
		R6/1 8mo	↓GM1, GD1a, GD1b, GT1b, GQ1b	Cb	HPTLC	Denny et al., 2010
		R6/2 4wo	↓GD1a	CC	Immunoblot	Di Pardo et al., 2016
		R6/2 6wo	↓GD1a, GT1b	CC	Immunoblot	
		R6/2 12wo	↓GM1, GD1a	St	Immunoblot, ChTxB	Di Pardo et al., 2016
		R6/2 12wo	↑GD1a, GT1b; ↓GM1	Cx	Immunoblot, ChTxB	Di Pardo et al., 2016
		R6/2 12wo	↓GM1, GD1a, GT1b	CC	Immunoblot, ChTxB	Di Pardo et al., 2016
AD	human	ave. 7.4y of dementia	↑GM2; ↓GM1, GD1a, GD1b, GT1b	FCx	HPTLC	Kracun et al., 1992a
		ave. 7.4y of dementia	↓GM1, GD1a, GD1b, GT1b	TCx	HPTLC	Kracun et al., 1992a
		ave. 7.4y of dementia	↓GM3, GM2, GM1, GD1a, GD3, GD1b, GT1b	Basal Telen.	HPTLC	Kracun et al., 1992a
		NR	↑GM2, GM1	FCx	TLC-ELISA	Molander-Melin et al., 2005
		NR	↑GM2; ↓GM1, GD1a, GD1b, GT1b	TCx	TLC-ELISA	Molander-Melin et al., 2005
	mouse	APPSL 24mo	↑GM3, GM2; ↓GT1a, GD3, GD1b,GQ1b	Cx	HPTLC	Barrier et al., 2007
		PSKi 3mo	↓GM3, GM2	Cx	HPTLC	Barrier et al., 2007
		PS1 M146L 12mo	↑GD1a; ↓GM3, GM2, GT1a	Cx	HPTLC	Barrier et al., 2007
		APPSL/PS1Ki 3mo	↑GM3, GM2; ↓GM1, GT1b, GD3	Cx	HPTLC	Barrier et al., 2007
		APPSL/PS1Ki 6mo	↑GM3, GM2, GD3; ↓GM1, GT1a	Cx	HPTLC	Barrier et al., 2007
		APPSL/PS1Ki 12mo	↑GM3	Cx	HPTLC	Barrier et al., 2007
		APPSL/PS1Ki 24mo	↑GM3	Cx	HPTLC	Barrier et al., 2007
		APPSL/PS1 M146L 12mo	↑GM3, GD1a, GD3; ↓GT1a	Cx	HPTLC	Barrier et al., 2007
	rat	APP21 12mo	↑GM3	Cx, Hip, WM	MALDI Imag.	Caughlin et al., 2018
		APP21 20mo	↓GD1	WM	MALDI Imag.	Caughlin et al., 2018
ALS	human	NR	↑GM2; ↓GD1b, GQ1b	FCx, TCx, MCx, PHG	HPTLC	Rapport et al., 1985
		NR	↑GD3; ↓GT1b	FCx, TCx, MCx	HPTLC	Rapport et al., 1985
		Early	↑GM1	CSF	HPLC	Henriques et al., 2017
		Advanced	↑GM1, GM3	Cervical spine	HPLC-MS	Dodge et al., 2015
	mouse	Early	↓GM1	Spinal cord, NMJ	HPLC-MS; IF	Dodge et al., 2015
		Early	↑GM3	Spinal cord	HPLC-MS	Dodge et al., 2015
		Advanced	↓GM1; ↑GM3	Spinal cord	HPLC-MS	Dodge et al., 2015
Stroke	rat	Hypoxia (newborn)	↓Gangliosides	Cerebral hemispheres	Bial’s Orcinol	Rastogi et al., 1968
		Hypoxia-ischemia (newborn)	↓GM1, GD1a, GD1b, GT1b	Hip	TLC-Resor.	Ramirez et al., 2003
		Bilateral intra-cerebral ventricular injection of Aβ and unilateral striatal injection of ET-1	↑GM3, GM2 ↓GM1, GD1	St	MALDI imag.	Caughlin et al., 2019
	mouse	MCAO	↑GM3, GM2	Hip, Cx, St, Tha, border of infarct tissue	MALDI Imag.	Whitehead et al., 2011
		MCAO	↑GM1, GD1a	Cx	MALDI Imag.	Whitehead et al., 2011
MS	human	NR	Inconsistent profiles	CSF	GO; HPTLC	Miyatani et al., 1990
		Remission and Relapse	Inconsistent profiles	CSF	HTPLC	Trbojevic-Cepe et al., 1991
		Remission	↑Gangliosides	Serum	TLC	Sela et al., 1982
		Remission and Relapse	↓Gangliosides	Plasma	TLC-GLC	Lebrun and Cherayil, 1976
		1^*st*^ relapse	↑GM1; ↓GM3	Serum	HPTLC	Zaprianova et al., 2001
		Relapse in long duration RRMS	↑GD1a; ↓GM1	Serum	HPTLC	Zaprianova et al., 2001

**Conditions*: AD, Alzheimer’s Disease; ALS, Amyotrophic Lateral Sclerosis; HD, Huntington’s Disease; HSP, Hereditary Spastic Paraplegia; MS, Multiple Sclerosis; PD, Parkinson’s Disease; S and P, Salt and Pepper Syndrome. *Stages:* NR, Not Reported; H and R, Hoehn and Yahr scale; NR, Not Reported; MCAO, Middle Cerebral Artery Occlusion; RRMS, Relapsing-Remitting Multiple Sclerosis. *Samples:* Cb, Cerebellum; Cb Cx, Cerebellar Cortex; CC, Corpus Callosum; Cx, Cortex; CSF, Cerebro-Spinal Fluid; FB, Forebrain; FCx, Frontal cortex; Hip, Hippocampus; MCx, Motor cortex; NMJ, Neuromuscular juncture Tibialis anterior muscle; OCx, Occipital cortex; PHG, Parahippocampal gyrus; SN, Substantia Nigra; St, Striatum; TCx, Temporal cortex; Telen., Telencephalon; Tha, Thalamus; WM, White Matter. *Methods:* ChTxB, Cholera Toxin B binding; IF, Immunofluorescence; GO, Glycolipid overlay; TLC, Thin Layer Chromatography; TLC-Resor., TLC-Resorcinol; HPTLC, High Performance TLC; HPTLC-Resor., HPTLC-Resorcinol; HPLC, High Performance Liquid Chromatography; NP-HPLC, Normal-phase HPLC; HPLC-MS, HPLC-Mass Spectrometry; MALDI, Matrix-assisted laser desorption/ionization Imaging; *Other:* ave, average; mo, months old; y, years; yo, years old; wo, weeks old.*

## Functions of Gangliosides in the Nervous System

Gangliosides modulate signaling by engaging in *cis-* and *trans-*interactions with a number of membrane receptors, adhesion molecules and ion channels. The specific nature of these interactions and their consequences (i.e., whether protein activity and signaling is increased or inhibited) depend on which gangliosides and proteins are involved. Some of the underlying mechanisms are general and apply to most gangliosides, while others are specific to distinct ganglioside headgroups.

### Ganglioside Modulation of Membrane Properties

Gangliosides display a natural propensity to laterally segregate and to associate with each other, with other sphingolipids and with cholesterol, through a network of hydrogen bonds and hydrophobic interactions (Sezgin et al., 2017). Changes in membrane ganglioside levels can indirectly affect membrane localization and function of a number of signaling proteins with glycosylphosphatidylinositol (GPI)- or palmitoyl-anchors, which display high affinity for gangliosides and sphingolipid-enriched membrane microdomains (Zhang et al., 1998).

Gangliosides are also important players in the formation of membrane domains with a strong positive curvature, which explains their enrichment at the growing tips of neurites, or at the edges of caveolae during caveolar invagination (Igarashi et al., 1994; Iwabuchi et al., 1998; Dasgupta et al., 2018). The biophysical effects of gangliosides on membrane curvature and other membrane properties is beyond the scope of this review, but the reader is referred to an excellent publication on this topic (Sonnino et al., 2018).

### *Cis-*Interactions of Gangliosides With Membrane Proteins

Gangliosides can directly bind membrane proteins via the establishment of carbohydrate-carbohydrate or carbohydrate-amino acid interactions (Yoon et al., 2006; Kabayama et al., 2007; Prasanna et al., 2016). Such interactions usually involve specific ganglioside headgroups and result in modulation of one or more of the following: (i) protein localization within membrane microdomains where recruitment of signaling partners occurs (Julien et al., 2013); (ii) receptor dimerization and/or (iii) receptor interaction with signaling partners and regulators (Kabayama et al., 2007; Julien et al., 2013). For example, interaction of GM3 with the epidermal growth factor receptor (EGFR) results in inhibition of receptor dimerization and activation (Yoon et al., 2006; Coskun et al., 2011). On the other hand, binding of GM1 to the tropomyosin-receptor kinase A (TrkA) promotes receptor dimerization and activation, and downstream outcomes such as neurite outgrowth and neuroprotection (Mutoh et al., 1995; Farooqui et al., 2002; Da Silva et al., 2005; Duchemin et al., 2008). Recent studies showed that the GM1 pentasaccharide headgroup is able to mimic the effects of the entire GM1 molecule (including the ceramide tail) on TrkA activation and signaling (Chiricozzi et al., 2017; Di Biase et al., 2020a). In contrast, both the glycan headgroup and the ceramide tail of GT1b are required for the interaction of this ganglioside with synaptotagmin 1/2 in order to form a high-affinity receptor complex for the botulinum toxin B at nerve endings (Flores et al., 2019).

A different mechanism of receptor activity modulation is involved in the interaction between GM3 and the insulin receptor. Increased levels of GM3 at the plasma membrane of adipocytes result in GM3 binding to a Lys residue in the extracellular domain of the insulin receptor (IR). This leads to the disruption of a crucial interaction between IR and caveolin, which, in turn, results in receptor internalization and inhibition of insulin signaling (Kabayama et al., 2007). Whether changes in ganglioside levels could also affect IR signaling in the brain is currently unknown. IR signaling regulates synaptic plasticity, neuronal survival and neurogenesis (Pomytkin et al., 2018). Both a decrease in brain IR signaling and an increase of GM3 levels were described in AD (Chan et al., 2012; Talbot et al., 2012), but whether the former is caused by the latter has not been investigated yet.

Another example of modulation of receptor turnover by gangliosides involves the GluR2-AMPA receptor (AMPAR) (Prendergast et al., 2014), an ionotropic glutamate receptor that is key to memory and learning processes (Hanley, 2014). AMPARs are abundant in GM1-enriched synaptic membranes (Cole et al., 2010). GluR2 AMPAR subunits can be pulled down from neuronal lysates using GM1-coated beads, suggesting an association between the receptor and the ganglioside (Prendergast et al., 2014). AMPARs internalization and removal from synaptic membranes requires the interaction with the AMPAR-trafficking complex (ATC) (Hanley, 2014). The latter normally binds GT1b, but it dissociates from this ganglioside in conditions that favor AMPAR internalization (Prendergast et al., 2014). The authors of this study proposed a model whereby binding of GluR2 and ATC to GM1 and GT1b, respectively, would result in their sequestration within distinct membrane microdomains and away from each other, thus ensuring that AMPARs are retained at the synaptic membrane. It was also suggested that such a mechanism might in part explain the cognitive impairment observed in mouse models and patients lacking complex gangliosides (Prendergast et al., 2014).

Finally, gangliosides can promote the formation of supramolecular complexes required for the integration of multiple cell signaling pathways, which often results in signal potentiation. A classic example is provided by the role of GM1 at the tip of neurites during axonal growth. By binding to TrkA receptors and laminin-1 and by clustering these molecules in the same membrane microdomains, GM1 brings together both TrkA and laminin-1/beta1 integrin signaling complexes and promotes axonal growth (Da Silva et al., 2005).

### *Trans-*Interactions and Co-receptor Role of Gangliosides

Gangliosides can interact *in trans* through their glycan moiety with proteins located on juxtaposed cell membranes or present in the extracellular space. The most relevant example of this type of interaction in the context of the CNS biology is the binding of the myelin-associated glycoprotein (MAG) on myelin sheaths to gangliosides present on axonal membranes. This interaction is crucial for the stability of myelinated axons (Lopez and Baez, 2018) and will be further discussed in the section “Role of Gangliosides in Neurite Outgrowth, Myelin Stability and Axon Structure.”

Gangliosides can also work as co-receptors (Fantini and Barrantes, 2009). The best characterized example is the role of GM1 as a co-receptor for the fibroblast growth factor 2 (FGF2), which is required for the activation of the FGF2 receptor in cells that lack heparan sulfate proteoglycans, the main FGF2 co-receptors (Rusnati et al., 2002). Recently, the α2,3-sialyllactose moiety of gangliosides GM3 and GM1 clustered within lipid rafts was shown to serve as a low-affinity receptor for soluble α-Klotho (Dalton et al., 2017), an endocrine factor with important neuroprotective, cognitive-enhancing and anti-aging properties (Cararo-Lopes et al., 2017). It remains unclear, however, whether the gangliosides might work in association with a yet to be identified protein receptor for α-Klotho.

Gangliosides bind with low affinity serotonin and other neurotransmitters containing amino groups (Price et al., 1979; Matinyan et al., 1989). It was proposed that gangliosides might affect neurotransmitter dynamics and efficiency by serving as co-receptors that “catch” such neurotransmitters when they are released at synapses, and route them toward their high-affinity receptors (Fantini and Barrantes, 2009). In support of this hypothesis, atomistic molecular dynamics simulation confirmed that serotonin, dopamine, melatonin, adenosine, epinephrine and norepinephrine, but not acetylcholine, bind to artificial model membranes bearing negatively charged lipids (Postila et al., 2016). Although this study did not directly address the role of gangliosides, these are the main negatively charged lipids in the outer leaflet of postsynaptic membranes. Later work showed that the presence of GM1 in artificial membranes enhances histamine and dopamine membrane binding (Juhola et al., 2018). The co-receptor function of gangliosides could be particularly important in the case of G-protein coupled receptors that have the neurotransmitter binding site buried within the membrane bilayer, since in this case the neurotransmitter molecules are required to first adhere to the membrane and then diffuse laterally to the receptor binding site (Postila et al., 2016).

Some of the proteins that bind gangliosides, including α-synuclein, amyloid beta and synaptotagmin, were shown to contain a glycosphingolipid binding motif (GBM) characterized by the presence of both basic and turn-inducing amino acid residues (Yahi and Fantini, 2014; Flores et al., 2019). The identification of this binding motif might facilitate the discovery of additional novel ganglioside protein interactors in the future.

### Role of GM1 in Ca^2+^ Homeostasis

Both physiological and pathological levels of gangliosides (as it occurs in lysosomal storage diseases) can significantly affect neuronal Ca^2+^ homeostasis and Ca^2+^ signaling by modulating the activity of several ion channels and transporters (Ledeen and Wu, 2015). Gangliosides might modulate voltage-dependent Ca^2+^ channel activity by altering membrane fluidity and/or by concentrating Ca^2+^ ions close to the channels through electrostatic interactions between negatively charged gangliosides and cations (Tanaka et al., 1997). Enrichment of the plasma membrane with GM1 resulting from the activity of neuraminidase 3 (NEU3) – a ganglioside-specific sialidase that converts GD1a and GT1b to GM1 – was shown to trigger Ca^2+^ influx in neuronal cells through T-type calcium channels, and to induce neuritogenesis (Wu and Ledeen, 1994). In contrast, polysialogangliosides increase, while GM1 decreases the activity of the plasma membrane Ca^2+^-ATPase (PMCA) (Jiang et al., 2014; Huang et al., 2015), a transporter that regulates neuronal Ca^2+^ homeostasis by exporting cytosolic Ca^2+^ to the extracellular environment (Garcia and Strehler, 1999).

Distinct mechanisms are involved in the ganglioside-dependent regulation of transient receptor potential channel 5 (TRPC5) and 6 (TRPC6). TRPC5 is activated upon GM1 and integrins α5β1 co-clustering at the plasma membrane, triggered by GM1 binding to galectin 1 or to multi-valent molecules such as cholera toxin B or IgM antibodies (Quattrini et al., 2001; Fang et al., 2002; Day and Kenworthy, 2015; Wu et al., 2016). On the other hand, the specific binding of soluble α-klotho to GM1 and GM3 within lipid rafts inhibits TRPC6 activity by blocking membrane insertion of the channel (Wright et al., 2019). Notably, TRPC6 has emerged as an attractive pharmacological target for its involvement in stroke, neuronal injury, seizure and neuropathic pain (Kim and Kang, 2015; Chen et al., 2017, 2019; Liu et al., 2020; Wang et al., 2020). Therefore, the inhibitory role played by gangliosides could be exploited therapeutically. To this end, TRPC6 inhibition was recently achieved by using a peptide that mimics the binding of α-klotho to gangliosides (Wright et al., 2019).

GM1 can also specifically regulate nuclear Ca^2+^ homeostasis. This function is mediated through high-affinity binding of the ganglioside to the sodium/calcium exchanger (NCX) at the nuclear envelope. Conversion of GD1a to GM1 by NEU3 at the nuclear envelope was shown to increase the local concentration of GM1, to promote the activity of NCX and to rapidly increase calcium efflux from the nucleus to the ER (Wang et al., 2009).

As expected by the large number of Ca^2+^ channels and transporters the activity of which is modulated by gangliosides, mice lacking complex gangliosides (GM2/GD2 synthase knock-out) have impaired Ca^2+^ regulation (Wu et al., 2001). Furthermore, pathological accumulation of GM2 in lysosomes and ER in a mouse model of Sandhoff disease, a lysosomal storage disorder caused by lack of β-hexosaminidase, results in reduced Ca^2+^ uptake by the SERCA pump and consequent ER stress, leading to neurite atrophy and apoptosis (Pelled et al., 2003; Virgolini et al., 2019). In a model of GM1 gangliosidosis, another lysosomal storage disease, GM1 accumulates in mitochondria-associated membranes (MAM) where it interacts with the IP3 receptor-1 and potentiates flux of Ca^2+^ into mitochondria, resulting in mitochondrial Ca^2+^ overload, mitochondrial membrane permeabilization and apoptosis (Sano et al., 2009).

### Role of Gangliosides in Apoptosis and Autophagy

Several studies have shown a direct role of ganglioside GD3 in apoptosis. GD3 synthesis is required for the induction of apoptosis upon cell treatment with FAS ligand (FASL), ceramide (De Maria et al., 1997), TNF (García-Ruiz et al., 2002) or Amyloid beta (Copani et al., 2002). Administration of exogenous GD3 (but not GD1a, GT1b or GM1) and overexpression of GD3 synthase were also shown to induce cell death (De Maria et al., 1997). The main mechanism by which GD3 induces apoptosis involves its redistribution from the Golgi apparatus or plasma membrane to mitochondrial membranes, where the ganglioside triggers the opening of the mitochondrial permeability transition pore (MPTP) (Kristal and Brown, 1999; Scorrano et al., 1999; García-Ruiz et al., 2000, 2002; Rippo et al., 2000). Furthermore, accumulation of GD3 in raft-like domains of the mitochondrial membrane promotes the recruitment of proteins required for mitochondrial fission and apoptosis [reviewed in Garofalo et al. (2015)]. Antioxidants and 9-*O*-acetylation of the GD3 molecule, a chemical modification that prevents ganglioside oxidation, block the pro-apoptotic effects of GD3 (Malisan et al., 2002; Colell et al., 2004), suggesting that products of GD3 oxidation might have stronger pro-apoptotic activity than GD3 itself (Brenner et al., 2010).

Increased GD3 levels were found in many neurodegenerative conditions (Table 1) and in primary cultures of cerebellar neurons maintained *in vitro*, which slowly undergo apoptosis (Copani et al., 2002). Whether the higher levels of GD3 are a predisposing factor and precede overt neurodegeneration, or whether they are simply a sign of ongoing apoptosis remains to be determined. GD3 levels also increase in reactive astrocytes (Kawai et al., 1994, 1999) as well as in activated microglia in human immunodeficiency virus (HIV) type 1 infections (Andersson et al., 1998). Of note, GD3 shed into the culture medium by activated microglia induces apoptosis in oligodendrocytes (Simon et al., 2002), and might affect neurons as well.

The effects of other gangliosides on apoptosis are likely indirect, mediated by the activation of pro-survival pathways (Mocchetti, 2005; Maglione et al., 2010), changes in Ca^2+^ homeostasis (Pelled et al., 2003; Virgolini et al., 2019) or other signaling events. This explains why the same ganglioside/s can have pro-apoptotic or anti-apoptotic effects depending on the context. As an example, while the b-series disialogangliosides GD3 and GD1b induce apoptosis in monocyte-derived dendritic cells and human breast carcinoma cells (Péguet-Navarro et al., 2003; Ma et al., 2004; Ha et al., 2016), upregulation of b-series gangliosides in neuroblastoma cells and in developing neurons is associated with increased protection from ceramide-induced apoptosis (Bieberich et al., 2001). As previously mentioned, pathological accumulation of GM1 or GM2 at the MAM or in the ER, respectively, triggers apoptosis in models of gangliosidosis (Pelled et al., 2003; Sano et al., 2009; Virgolini et al., 2019). In contrast, administration of exogenous GM1 exerts anti-apoptotic effects in neuronal cells exposed to a variety of stress and toxic conditions (Mocchetti, 2005; Maglione et al., 2010). We speculate that a potential explanation for the discrepancy between these studies and the former might be related to differences in the intracellular traffic and distribution of the exogenously administered ganglioside compared to the endogenous gangliosides that accumulate in lysosomal storage diseases.

In addition to its prominent role in apoptosis, GD3 is also a regulator of autophagy, a process that is central to proteostasis and to the degradation and/or recycling of cellular components (Tommasino et al., 2015). Inhibition of sphingolipid synthesis or knock-down of GD3 synthase inhibited autophagy in human fibroblasts, an effect that was reversed by treatment with GD3 (Matarrese et al., 2014). GD3 is present in autophagosome membranes where it colocalizes with key players in autophagosome biogenesis and maturation, including LC3, PI3P and LAMP1 (Matarrese et al., 2014). Furthermore, the interaction of the autophagy initiator protein AMBRA1 with the ER chaperone calnexin, which is required for the formation of autosomal membranes, was shown to depend on the presence of GD3 in raft-like domains of the MAMs, and was disrupted by the knock-down of GD3 synthase (Garofalo et al., 2016).

The role of other gangliosides in autophagy is less clear and likely indirect. As it is the case for apoptosis, both inhibitory (Chung et al., 2010; Hwang et al., 2010; Li et al., 2016) and stimulatory effects (Wei et al., 2009a; Meng et al., 2016; Dai et al., 2017; Guo et al., 2020) of gangliosides on autophagy have been reported. Inhibition of ganglioside synthesis was shown to suppress autophagy, causing accumulation of α-synuclein and cell death in a neuroblastoma model of PD. Addition of a mix of several gangliosides, including GD3, restored both autophagy and cell viability (Wei et al., 2009a). Whether these effects were due to GD3 only or to other gangliosides remains unclear. Administration of GM1 alone was shown to activate autophagy *in vitro* and *in vivo*, resulting in increased clearance of α-synuclein in PD cells (Guo et al., 2020) and decreased apoptosis in AD models (Dai et al., 2017). However, the conclusions from these studies are not generalizable. In fact, in a different context (a rat model of stroke), GM1 had the opposite effect and provided neuroprotection by inhibiting autophagy, rather than activating it.

### Role of Gangliosides in Neurite Outgrowth, Myelin Stability and Axon Structure

Several studies have shown neuritogenic activity of exogenously administered (Roisen et al., 1981; Facci et al., 1984; Skaper et al., 1985) or endogenously produced GM1 (Da Silva et al., 2005) *in vitro*. In hippocampal cultures, focal generation of GM1 by NEU3 was shown to be required for the establishment of neuronal polarity and the development of a leading axon from multiple neurites. The underlying mechanism involves focal enhancement of TrkA activity, inhibition of RhoA signaling and local actin depolymerization (Da Silva et al., 2005).

GD1a and GT1b – the most abundant gangliosides in axonal membranes (De Vries and Zmachinski, 1980) – are major ligands for MAG (also known as siglec-4) (Yang et al., 1996), a sialic acid-binding lectin expressed by myelinating oligodendrocytes in the CNS and in Schwann cells in the peripheral nervous system (PNS) (Quarles, 2007). The 0-series gangliosides GM1b and GD1α (Figure 1) can also bind MAG with high affinity and compensate for the lack of a- and b-series gangliosides in mouse models (Collins et al., 1999). The interaction between MAG and axonal gangliosides confers structural stability to myelinated axons and provides protection against axonal toxicity induced by neurotoxins (Pan et al., 2005; Mehta et al., 2010). These effects appear to be mediated by the activation of the RhoA/Rock pathway and the tubulin polymerization factor CRMP4, and by the stabilization of axonal neurofilaments and microtubules (Nguyen et al., 2009; Nagai et al., 2012). In axonal membranes, GT1b is found in association with a multimeric signaling complex comprising the Nogo-66 receptor NgR1, Lingo-1 and the neurotrophin receptor p75NTR. Binding of MAG to this complex and to GD1a and GT1b results in inhibition of axon outgrowth, which prevents unwanted axonal sprouting in healthy conditions, as well as also repair after injury of the CNS (Vinson et al., 2001; Williams et al., 2008; Saha et al., 2011; Lopez and Baez, 2018). Of note, upon axotomy, activation of NEU3 in PNS, but not in CNS axons, results in the conversion of the inhibitory gangliosides GD1a and GT1b to GM1, which relieves inhibitory signals in the PNS. This might explain, at least in part, why PNS but not CNS axons have the ability to regenerate (Kappagantula et al., 2014). The major role played by gangliosides in ensuring myelin stability is evident in *B4galnt1*-null mice, where the absence of ganglioside ligands for MAG results in decreased central myelination, peripheral dysmyelination, impaired nerve conduction and axonal degeneration (Sheikh et al., 1999; Pan et al., 2005; Susuki et al., 2007). A similar phenotype is shown by *Mag*-null mice (Fruttiger et al., 1995) and by patients with hereditary spastic paraplegia caused by mutations in the *MAG* or in the *B4GALNT1* gene (Roda et al., 2016; Trinchera et al., 2018).

Gangliosides are crucial for the organization of nodes and paranodes in myelinated fibers, and to ensure proper compartmentalization of adhesion molecules essential for the correct cytoarchitecture of paranodal regions, including neurofascin-155 (NF-155) and contactin/caspr1 (Susuki et al., 2007). Furthermore, gangliosides associate with and play a key role in the compartmentalization of Kv1 channels and their anchoring proteins – CASPR2 and transient-axonal glycoprotein 1 (TAG-1) – in juxtaparanodal regions (Kasahara et al., 2000; Susuki et al., 2007). In *B4galnt1-*null mice, nodes of Ranvier and paranodes are disorganized and Kv1.1 and Na^+^ channels are mislocalized (Susuki et al., 2007). Although it was proposed that *axonal* gangliosides may play a lion’s share in maintaining nodal and paranodal structure (Yao et al., 2014), in the PNS, *myelin* gangliosides cooperate with the former for the organization of paranodal and juxtaparanodal regions (Kleinecke et al., 2017).

## Gangliosides in Development and Aging

Expression of ganglioside biosynthetic enzymes and production of specific gangliosides are developmentally regulated processes (Yanagisawa and Yu, 2007; Itokazu et al., 2018). At early developmental stages, in the rodent brain, the ganglioside profile is dominated by simple gangliosides (GM3 and GD3). GD3 is the predominant ganglioside expressed on the surface of neural stem cells (NSC) where its interaction with the EGFR ensures receptor recycling after endocytosis and maintenance of self-renewal capacity (Itokazu et al., 2018). NSC differentiation to neuronal progenitors is accompanied by a shift from the synthesis of GD3 to GM1, which was proposed to promote expression of neuronal-specific genes (Itokazu et al., 2017). At later developmental stages, complex gangliosides (GM1, GD1a, GD1b, and GT1b) become the predominant fraction throughout the brain (Ngamukote et al., 2007) and remain relatively constant during adulthood. Changes, however, might occur with aging.

In a study that examined the ganglioside composition of 118 brains from healthy subjects covering an age range from 20 to 100 years, a curvilinear relationship between age and ganglioside levels was observed in the gray matter of frontal and temporal cortex: gangliosides increased modestly from 20 to 50 years of age and then decreased slightly, with a more rapid decline starting at 70 years of age. The overall drop in the concentration of total gangliosides was 11% in frontal and 18% in temporal cortex (Svennerholm et al., 1994). An age-related decline in ganglioside concentration in the frontal, but not the occipital cortex was described in a second report (Kracun et al., 1991). Determining what causes these changes is not always easy. A global reduction of total ganglioside levels that is accompanied by similar changes in phospholipid and cholesterol (Svennerholm et al., 1994) might merely reflect an altered brain cytoarchitecture (e.g., increase in glia cells, loss of synapses, etc.), cell death or tissue atrophy. On the other hand, a shift in the ganglioside profile might reveal metabolic and/or cellular alterations. In regard to this, an age-related relative decrease in GD1a and to a lesser extent GM1, and a relative increase of GD1b, GM3, and GD3 was reported in healthy subjects (Svennerholm et al., 1994). Furthermore, a study that analyzed ganglioside levels in the substantia nigra of 20 healthy subjects confirmed a decrease in total gangliosides after the age of 60 with a specific reduction in GM1 and GD1a (Huebecker et al., 2019). A decrease in GD1a, GD1b, and GT1b (either in total brain or myelin fractions) was also confirmed in aged mice compared to young animals, concomitantly with increased levels of ganglioside precursors (lactosylceramide and glucosylceramide), suggesting a block in the biosynthesis of gangliosides. Differently from humans, however, GM1 levels were generally increased or not changed in aged mice (Ohsawa, 1989; Palestini et al., 1993; Waki et al., 1994; Yamamoto et al., 2008; Hallett et al., 2018). Overall, the available evidence suggests that a specific alteration of the brain ganglioside profile occurs with aging, with a shift from complex to simple gangliosides. Of note, studies in mice have also revealed a relative age-dependent and brain region-specific increase in ganglioside species with a longer sphingosine moiety (d20:1) (Sugiura et al., 2008; Caughlin et al., 2017). These observations are in line with the report of an overall increase in the levels of sphingosine d20:1, which was proposed to be a metabolic marker of aging in the brain (Palestini et al., 1990). Whether similar ganglioside changes occur in the aging human brain remains to be determined. As methods for the analysis of gangliosides in the brain become more sensitive and refined (Dehelean et al., 2019; Andres et al., 2020), so will our understanding of the extent to which the normal aging process affects brain gangliosides.

## Gangliosides in Neurological and Neurodegenerative Conditions

The crucial role of gangliosides in CNS homeostasis is highlighted by the fact that both lack and excess of gangliosides result in severe neurodegenerative conditions. On one hand, accumulation of gangliosides due to mutations in lysosomal degradative enzymes causes fatal lysosomal storage diseases such as GM1-gangliosidosis, Tay-Sachs and Sandhoff diseases, which are characterized by a broad range of clinical symptoms, developmental delay and early onset neurodegeneration (Annunziata et al., 2018; Platt et al., 2018). On the other hand, mutations that block ganglioside synthesis result in severe early onset epileptic syndromes and neurodegeneration (Trinchera et al., 2018). While these are pathological extremes, growing evidence suggests that more subtle changes in ganglioside levels and profile (i.e., in the relative abundance of specific gangliosides) might occur in more common pathological conditions, including PD, HD, AD, amyotrophic lateral sclerosis (ALS) and multiple sclerosis (MS) (Table 1), in some cases significantly contributing to pathology.

### Diseases Resulting From Major Defects of Ganglioside Biosynthesis

Mutations in the *ST3GAL5* gene, which codes for GM3 synthase, results in lack of all gangliosides in humans. Patients who carry rare mutations in this gene develop the infantile-onset symptomatic epilepsy syndrome, and present with severe, refractory epilepsy accompanied by neurological deterioration that starts in the first few years of life and leads to motor and cognitive impairment (Simpson et al., 2004; Fragaki et al., 2013; Boccuto et al., 2014). Dyspigmentation of the skin and abnormal auditory responses are also present in some patients (Boccuto et al., 2014; Yoshikawa et al., 2015). None of these features – except for hearing impairment due to the degeneration of stereocilia in the hair cells of the organ of Corti – is reproduced in *St3gal5*-null mice (Yoshikawa et al., 2015). The explanation for this interspecies difference might lie in the fact that the murine B4GALNT1 (GM2/GD2 synthase, the enzyme downstream of ST3GAL5 in the ganglioside biosynthetic pathway, Figure 1) can overcome the metabolic block by using lactosylceramide as a substrate for the synthesis of the 0-series gangliosides GM1b and GD1α and compensate for the lack of a- and b-series gangliosides. On the contrary, the human B4GALNT1 ortholog cannot (Yamashita et al., 2003). Patients with ST3GAL5 loss of function present with additional biochemical anomalies compared to mice, including an increase in the levels of globosides (another class of glycolipids) (Liu et al., 2008; Fragaki et al., 2013; Boccuto et al., 2014) and an altered pattern of protein glycosylation (Nagahori et al., 2013), both of which might contribute to the more severe phenotype presented by patients compared to mouse models (Yamashita et al., 2003; Yoshikawa et al., 2015).

Loss-of-function mutations in the *B4GALNT1* gene cause a block of ganglioside biosynthesis downstream of GM3 and GD3 (Bhuiyan et al., 2019) and increased GM3 levels (Harlalka et al., 2013), resulting in a complicated form of hereditary spastic paraplegia (HSP26). This suggests that GM3 cannot compensate for the lack of complex gangliosides. HSP26 is clinically characterized by spasticity of lower limbs and intellectual disability, which can be accompanied by cortical atrophy, peripheral neuropathy, psychiatric or endocrinological problems depending on the specific mutation inherited (Boukhris et al., 2013; Harlalka et al., 2013; Wakil et al., 2014). Mutations that partially preserve B4GALNT1 activity result in milder symptoms and later onset (Bhuiyan et al., 2019), suggesting a direct correlation between the extent of ganglioside reduction and disease severity. Demyelination, motor and sensory dysfunctions are likely the result of a disrupted interaction between MAG and the axonal gangliosides GD1a and GT1b, as these aspects of the disease are phenocopied in patients carrying MAG mutations (Novarino et al., 2014; Lossos et al., 2015; Roda et al., 2016). *B4galnt1*-null mice mirror the human pathology and display motor and sensory problems due to demyelination and axonal degeneration (Takamiya et al., 1996; Sheikh et al., 1999; Chiavegatto et al., 2000; Sugiura et al., 2005), and deficits in cognition and hippocampal plasticity (Sha et al., 2014).

### Epilepsy

Epilepsy is a complex neurological disorder characterized by seizures due to aberrant and asynchronous neuronal activity in the brain. The underlying etiology can vary from genetic defects to developmental problems, infections or trauma (Behr et al., 2016). Since severe epilepsy is associated with defects in ganglioside synthesis in humans, and audiogenic seizures are a feature of *St3gal5*-null mice (Kawai et al., 2001), the question arises whether changes in brain ganglioside levels could underlie or contribute to other forms of epilepsy. Decreased ganglioside levels, especially GM1 and GD1a were found in the cerebro-spinal fluid (CSF) of a small number of patients with West syndrome (Izumi et al., 1993), an infantile epilepsy disorder caused by a variety of genetic, metabolic and developmental factors (D’Alonzo et al., 2018). More recently, loss-of-function mutations in ST3GAL3 – one of the two sialyltransferases involved in the synthesis of GD1a and GT1b (Sturgill et al., 2012; Figure 1) – were found in patients with West syndrome (Edvardson et al., 2013) and in two siblings with epileptic encephalopathy (Indellicato et al., 2019). In animal models, ST3GAL3 catalyses the sialylation of glycoproteins in addition to glycolipids (Sturgill et al., 2012). However, the pattern of protein glycosylation is only marginally affected in neurons derived from human induced pluripotent stem cells (iPSCs) from a patient, suggesting that lack of gangliosides, rather than aberrant protein glycosylation, could be the main cause of epilepsy in West patients with ST3GAL3 mutations (van Diepen et al., 2018).

Decreased hippocampal levels of the major brain gangliosides were also found in a pilocarpine-induced mouse model of epilepsy (de Freitas et al., 2010). In amygdaloid kindling-mice, a model of temporal lobe epilepsy, increased brain levels of GQ1b increased brain levels of GQ1b compared to wild-type mice were reported, concomitantly with a reduction in the levels of GM1, leading to the speculation that high GQ1b levels could cause seizures in these mice (Kato et al., 2008). Intraventricular infusion of a mix of brain gangliosides (which would contain GQ1b) worsened kindling seizure (Nakamura et al., 1989), while administration of GM1 (Fighera et al., 2006) or GT1b (Yamamoto and Mohanan, 2003) alone protected animals from convulsions induced by injection of glutaric acid or kainic acid into the striatum, respectively. A clear mechanistic understanding of the role of gangliosides in epilepsy is still missing, but their ability to modulate the activity and trafficking of membrane receptors and ion channels, including GluR2 AMPAR subunits (Prendergast et al., 2014), is likely to be involved.

### Parkinson’s Disease

PD is a neurodegenerative disease in which patients experience motor and non-motor deficits due to degeneration of dopaminergic neurons mainly in the substantia nigra. In most forms of PD, patients present with insoluble deposits of protein aggregates primarily composed of α-synuclein (Poewe et al., 2017).

Several studies have linked altered ganglioside levels and parkinsonism in mouse models and patients. *B4galnt1*^–/–^ mice display PD-like motor impairments, concomitant with degeneration of tyrosine hydroxylase (TH)-positive neurons and increased accumulation of α-synuclein in the substantia nigra (Wu et al., 2011). Even a partial reduction of ganglioside levels in heterozygous *B4galnt1*^+/–^ mice produces a similar phenotype, although milder, and results in short-term memory loss, heart and colon lesions as well as symptoms of constipation – all of which are characteristic of PD (Chiavegatto et al., 2000; Wu et al., 2012, 2020). Administration of L-DOPA (Wu et al., 2011, 2012), GM1 (Wu et al., 2020), or a semisynthetic lysoderivative of GM1, Liga20, decreased symptoms and neuropathology in these mice (Wu et al., 2011, 2012, 2020). These studies suggest that depletion of gangliosides may be upstream of PD molecular pathology and contribute to PD pathogenesis. In further support of this hypothesis, pharmacological inhibition of ganglioside synthesis exacerbates the lysosomal dysfunction induced by α-synuclein in a neuroblastoma cell line (Wei et al., 2009b). Furthermore, knockdown of *B3GALNT4* in a dopaminergic cell line to reduce GM1 levels increases cell sensitivity to MPP^+^-induced toxicity (Verma and Schneider, 2019). On the other hand, experimentally increasing GM1 levels is protective in both chemical and α-synuclein-induced models of PD (Schneider et al., 1992, 2019; Wei et al., 2009b; Guo et al., 2020). Knock-down or deletion of *St8sia1* (GD3 synthase) is also protective in the MPTP model of PD, likely due to accumulation of GM1 that occurs in these models when GM3 cannot be channeled for the synthesis of GD3 (Figure 1; Akkhawattanangkul et al., 2017; Dhanushkodi et al., 2019).

A link between ganglioside levels and PD is also supported by human studies. GD1a and GT1b were shown to be decreased in the substantia nigra of male PD patients compared to healthy subjects (Seyfried et al., 2018). A second study on 30 post-mortem substantia nigra samples from sporadic PD patients and 15 age-matched controls confirmed the decrease of GD1a and GT1b in PD patients, along with decreased levels of GM1 and GD1b, and a concomitant increase in the ganglioside precursor glucosylceramide (Huebecker et al., 2019). These changes were recapitulated in PD CSF samples (where GM2, GD3, GD1a, GD1b, GT1b as well as total ganglioside levels were significantly decreased) and, partially, in PD serum (where only GM1 and GD1a were decreased) (Huebecker et al., 2019). Interestingly, the simple ganglioside GM3 was found to be increased in PD CSF compared to controls (Huebecker et al., 2019), in line with a similar shift from complex to simple gangliosides observed in aging brains and in AD models (Kracun et al., 1992a; Svennerholm et al., 1994; Caughlin et al., 2018). Alterations in ganglioside metabolism may predate neuronal loss in PD, as suggested by the fact that the mRNA expression of the ganglioside biosynthetic enzymes B3GALT4 (GM1 synthase) and ST3GAL2 (GD1a/GT1b synthase) and the levels of GM1 are lower than normal in residual dopaminergic neurons in the substantia nigra of PD patients (Wu et al., 2012; Schneider, 2018).

Alpha-synuclein is a primary target in the treatment of PD. Treatment with GM1 was recently shown to increase α-synuclein clearance in a cell model of PD, by stimulating autophagy (Guo et al., 2020). In another study, administration of GM1 resulted in smaller α-synuclein aggregates *in vivo*, in a rat α-synuclein model of PD (Schneider et al., 2019), likely due to a direct effect of GM1 on α-synuclein folding and aggregation. A glycosphingolipid binding motif containing a critical tyrosine residue (Y39) was identified in α-synuclein, which allows interaction of the protein with gangliosides (GM3 > Gb3 > GM1 > GM4 > GM2 > asialo-GM1 > GD3) (Fantini and Yahi, 2011). GM1-containing vesicles interact with α-synuclein and promote a helical folding, delaying or even abolishing the formation of α-synuclein fibrils, especially if α-synuclein is acetylated at the N-terminus, as it normally occurs in cells (Anderson et al., 2006; Bartels et al., 2014). Altogether, the data discussed above suggest that GM1 might be a disease-modifying therapy for PD, one that is able to prevent aggregation of endogenous α-synuclein and promote its cellular clearance.

### Huntington Disease

HD is a protein-misfolding neurodegenerative disease that presents with a triad of motor, cognitive and psychiatric symptoms. In the juvenile form of the disease, seizures are also commonly present (Rosas et al., 2003; Bates et al., 2015). HD is caused by a mutation in the *HTT* gene which results in the expansion of a polyglutamine stretch (polyQ) near the N-terminus of the huntingtin protein (HTT) and consequent mutant HTT (mHTT) misfolding and aggregation (Roos, 2010). N-terminal proteolytic cleavage products of mHTT, as well as a protein fragment encoded by the first exon of the *HTT* gene and generated by aberrant splicing, are most toxic and prone to aggregate (Bates et al., 2015). Expression of mHTT causes widespread cellular dysfunctions and neuronal death mainly in the striatum and in the cortex (Bates et al., 2015). Similar to other amyloidogenic proteins, the interaction of mHTT with lipid membranes promotes its aggregation and fibrillation and can result in membrane disruption and permeabilization that contribute to pathogenesis (Burke et al., 2013).

Prior to the identification of the *HTT* gene and the establishment of rigorous diagnostic criteria for the disease, alterations in brain gangliosides were reported in post-mortem tissue from patients with presumed HD (Hooghwinkel et al., 1968; Bernheimer et al., 1979; Higatsberger et al., 1981). Later studies revealed that downregulation of ganglioside biosynthetic enzymes occurs in the brain of genetic HD mouse models expressing full-length (YAC128 mice) (Maglione et al., 2010), or exon 1 mHTT (R6/1 mice) (Desplats et al., 2007), and in fibroblasts from HD patients (Maglione et al., 2010). Accordingly, levels of GM1 were found to be significantly reduced in skin fibroblasts from HD patients (Maglione et al., 2010) and in affected brain regions in mouse models (Desplats et al., 2007; Maglione et al., 2010; Di Pardo et al., 2016). GD1a and GT1b were also found to be decreased in the YAC128 model (Maglione et al., 2010). In three human HD caudate nucleus samples, an average 40% reduction of total ganglioside levels was found, concomitantly with a significant increase in GD3 levels and a trend toward decreased GM1 levels (Desplats et al., 2007). On the contrary, GM1 levels were increased in HD cerebellum, a brain region that is relatively spared in HD (Denny et al., 2010; Cloud et al., 2012). It is not possible to determine whether decreased ganglioside levels in the HD caudate nucleus occur prior to or as a consequence of the neurodegenerative process. However, the finding that ganglioside metabolism and GM1 levels are decreased in primary human fibroblasts as well as in YAC128 mice prior to neurodegeneration and loss of tissue (Maglione et al., 2010) clearly suggests that impairment of ganglioside synthesis is independent from and predates neurodegenerative changes in HD. Interestingly, a deficit in ganglioside GM1 was detected in primary cultures of YAC128 neurons, but not in astrocytes, which are more resilient to the toxic effects of mHTT (Maglione et al., 2010).

Decreased levels of GM1 in neuronal HD cells was shown to contribute to heighten HD cell susceptibility to growth factor deprivation. Restoring normal cellular levels of GM1 by administration of exogenous ganglioside resulted in protection from cell death. Furthermore, when the synthesis of gangliosides was decreased in wild-type cells using an inhibitor of the ganglioside biosynthetic pathway, these cells became as susceptible to growth factor deprivation as HD cells, suggesting that cellular levels of GM1 modulate the ability of neuronal cells to cope with the removal of pro-survival signals. Contrary to the expectations, this neuroprotective role of GM1 was not dependent on activation of Trk receptors, the best characterized targets for exogenous GM1 (Mocchetti, 2005), and only partially depended on the activation of the neuronal pro-survival PI3K/AKT pathway (Maglione et al., 2010), suggesting that additional protective mechanisms, which will be discussed later, are activated by GM1 administration in HD models.

Restoration of ganglioside levels by administration of exogenous GM1 in HD mice by chronic intraventricular infusion resulted in dramatic therapeutic and disease-modifying effects, with reversal of motor, cognitive and psychiatric-like symptoms, slowdown of neurodegeneration and myelin atrophy, and normalization of neurotransmitters levels (Di Pardo et al., 2012; Alpaugh et al., 2017). Such widespread therapeutic effects can only be explained if, in addition to established neuroprotective pathways, GM1 can also target mHTT directly. Indeed, GM1 administration significantly decreased brain levels of soluble and aggregated mHTT, without affecting HTT transcription or wild-type HTT levels, suggesting that GM1 might promote mHTT clearance at a cellular and/or systemic level (Alpaugh et al., 2017). The underlying mechanisms remain to be determined, although it was found that GM1 treatment increases phosphorylation of mHTT at Ser13 and Ser16, a crucial post-translational modification associated with a reduction in mHTT aggregation and toxicity (Gu et al., 2009). Furthermore, a recent study showed that the presence of GM1 in artificial membranes made with total brain lipid extract dramatically decreases membrane insertion of exon 1 mHTT, and slows down the appearance of mHTT oligomers formed on membranes (Chaibva et al., 2018). Whether administration of GM1 can affect the kinetics of mHTT aggregation or the aggregate species that are formed *in vivo* remains unknown.

### Alzheimer’s Disease

AD is the most prevalent neurodegenerative disease and is characterized by production, misfolding and aggregation of Aβ, as well as aggregation of hyperphosphorylated microtubule protein tau in neurofibrillary tangles (Masters et al., 2015).

Various studies suggested the presence of altered brain ganglioside levels in post-mortem brains from AD patients (Op Den Velde and Hooghwinkel, 1975; Crino et al., 1989; Kracun et al., 1992a). Ganglioside levels were shown to be decreased in a region-specific manner, while an increase in simple ganglioside species such as GM2, GM3, and GM4 was observed in the frontal cortex of patients (Kalanj et al., 1991; Kracun et al., 1992a). These changes were mirrored by recent findings in the Tg APP21 rat model of AD, where the relative abundance of simple gangliosides, in particular GM3, increases by 12 months of age in various brain regions, while d18:1 GD1 species decrease by 20 months of age (Caughlin et al., 2018). In contrast, a different study showed no changes in ganglioside levels in the frontal cortex of AD patients compared to healthy controls, but a specific enrichment of GM1 and GM2 was observed in detergent-resistant membranes, suggesting an alteration of lipid microdomains that might have an impact on signaling (Molander-Melin et al., 2005). In the hippocampus and other affected brain regions of AD mice, gangliosides with long chain bases (d20:1) were shown to increase early on, leading to the hypothesis that they could be involved in AD dysfunctions (Hirano-Sakamaki et al., 2015; Caughlin et al., 2018; Kaya et al., 2020). The underlying cause of altered ganglioside content in AD brains have yet to be elucidated. Proliferation of astrocytes (astrogliosis) that skews the ganglioside profile, or increased catabolism of gangliosides in AD patients have been proposed to explain the data obtained in human tissue (Kalanj et al., 1991; Kracun et al., 1992a). Indeed, fibroblasts from AD patients catabolize GM1 at a higher rate than control fibroblasts *in vitro* (Pitto et al., 2005).

The overall role of gangliosides, especially GM1, in AD pathogenesis is controversial. Early studies found GM1 bound to Aβ in post-mortem AD and Down-syndrome brains. It was proposed that this GM1-bound Aβ might work as a seed for Aβ aggregation (Yanagisawa et al., 1995). A large body of literature showed that GM1 binding to Aβ promotes the amyloidogenic process, a property shared by other gangliosides as well, depending on the specific variants of Aβ used in the experiments (Matsuzaki, 2020). Unilamellar vesicles containing GM1 increase the rate of Aβ fibrillization *in vitro* (Choo-Smith et al., 1997), by inducing a β-sheet structure in the peptide with a high propensity to aggregate (Matsuzaki and Horikiri, 1999; Kakio et al., 2001). Of note, the isolated pentasaccharide of GM1 cannot bind Aβ, suggesting the interaction requires a polyanionic surface, as provided by clusters of gangliosides on membranes (Williamson et al., 2006). Gangliosides bearing more sialic acid residues display higher affinity for Aβ (Matsuzaki and Horikiri, 1999; Ariga et al., 2001), but induce formation of Aβ fibrils at a slower rate and less extensively than unilamellar vesicles containing GM1 (Kakio et al., 2002). Notably, oligomers of Aβ forming in aqueous solution display a different secondary structure and organization than those formed in the presence of ganglioside-containing membranes (Matsubara et al., 2018). The overall lipid environment in which GM1 is presented is also crucial for Aβ fibril formation (Kakio et al., 2001).

Extrapolation of *in vitro* findings to *in vivo* systems must be done with caution. A caveat of most *in vitro* studies is represented by the high concentrations of gangliosides and Aβ used, which might exceed physiological levels (Amaro et al., 2016). At more physiological concentrations, GM1 was shown to form microdomains in artificial membranes, and did not induce oligomerization of Aβ (Amaro et al., 2016). Thus, the amyloidogenic properties of GM1 *in vitro* remain controversial (Matsuzaki, 2020).

*In vivo* studies do not support the amyloidogenic role of GM1 in AD and, in some cases, even show beneficial and protective effects of this and perhaps other gangliosides as well. Knockout of *St8sia1* (GD3 synthase) in a transgenic mouse model of AD (APP/PSEN1) resulted in lack of b-series gangliosides and a concomitant increase of a-series gangliosides, including a >50% increase in GM1 (Bernardo et al., 2009). In spite of this, accumulation of Aβ plaques was decreased and behavior improved compared to mice with intact GD3 synthase (Bernardo et al., 2009). This suggests that either b-series gangliosides contribute to AD pathology, or the increase of a-series gangliosides and GM1 is protective. Similar protection from AD pathology was achieved by knocking out *St3gal5* (GM3 synthase) in the 5XFAD mouse model of AD, which resulted in the lack of both a- and b-series gangliosides (Dukhinova et al., 2019). Although these data could suggest that b-gangliosides are detrimental in AD mouse models, as their ablation was common to both models with improved outcomes, another possible explanation is that increased compensatory synthesis of 0-series gangliosides (Yamashita et al., 2003) offered protection in the absence of a- and b-series gangliosides. In a third study, *B4galnt1* (GM2 synthase) knockout mice were crossed with 1XFAD mice to generate a line of AD mice lacking GM1 and other complex a-, b- and 0-series gangliosides. In the brain of these mice, Aβ plaque burden was increased (Oikawa et al., 2009). In summary, data from different animal models point at a protective role of a-series and 0-series gangliosides *in vivo* in AD models. In support of this hypothesis, administration of exogenous GM1 protects mice and rats from Aβ-induced toxicity and attenuates the resulting cognitive deficits (Yang et al., 2013; Dai et al., 2017), by increasing autophagic clearance of Aβ (Dai et al., 2017), reducing oxidative stress (Yang et al., 2013), and/or through binding and scavenging of Aβ (Huang et al., 2015). Infusion of GQ1b was also beneficial in an AD mouse model, by increasing BDNF levels, reducing Aβ plaque levels, decreasing tau phosphorylation and rescuing cognitive impairments (Shin et al., 2019).

### Amyotrophic Lateral Sclerosis

ALS is a fatal neurodegenerative disease characterized by degeneration of upper and lower motor neurons (Hardiman et al., 2017). A few studies suggested impaired ganglioside metabolism as part of the pathophysiology of ALS (Dodge et al., 2015; Henriques et al., 2015, 2017; Bouscary et al., 2019). Elevated GM2 and GD3 and decreased GD1b, GT1b, and GQ1b levels were found in the brain of ALS patients compared to healthy controls (Rapport et al., 1985). In a different study, GM1 and GM3 were shown to be significantly elevated in the spinal cord, together with the levels of enzymes that catalyze glycosphingolipid hydrolysis, such as non-lysosomal glucosylceramidase and hexosaminidase (Dodge et al., 2015; Bouscary et al., 2019), suggesting a potential impairment of the catabolism of gangliosides in ALS. In addition, a positive correlation between the levels of GM1 in the CSF and the severity of the disease was described (Henriques et al., 2017). Anti-ganglioside antibodies were also detected in serum as well as CSF of ALS patients (Stevens et al., 1993; Niebroj-Dobosz et al., 1999; Kollewe et al., 2015), but their contribution to the pathogenesis of ALS is controversial (Adams et al., 1991; Kollewe et al., 2015).

In contrast to the findings in ALS patients, SOD1^*G93A*^ ALS mice showed reduced GM1 levels compared to wild-type mice in both early symptomatic and end stages of the disease (Dodge et al., 2015; Henriques et al., 2017). Data from different studies showed that treatments that increase ganglioside levels in ALS mouse models – including administration of exogenous GM3 (Dodge et al., 2015) and inhibition of the ganglioside catabolic enzymes glucosylceramidase beta 2 (Bouscary et al., 2019) and acid β-glucosidase (Henriques et al., 2017) – attenuated disease manifestation, slowed down disease progression and prolonged mouse survival. Interestingly, a single intraperitoneal administration of recombinant natural human IgM (rHIgM12) immunoglobulins against GD1a and GT1b increased survival in two different SOD models (SOD1^*G86R*^ and SOD1^*G93A*^), and delayed neurological deterioration (Xu et al., 2015). The authors of this study suggested that this treatment may block MAG-induced inhibition of axonal growth and repair, allowing neurons to regenerate after they incur damage. An alternative explanation is that antibody-mediated clustering of GD1a and GT1b might alter membrane microdomains and activate protective signaling pathways (Xu et al., 2015).

### Stroke

In the United States, stroke is the fifth leading cause of death. The incidence of stroke, which can be ischemic or hemorrhagic and commonly associated with hypoxia (Hankey, 2017), increases with age (Writing Group Members et al., 2010).

Induction of hypoxia results in a reduction in the levels of gangliosides in rat and human neonatal and premature brains (Qi and Xue, 1991). Furthermore, it was shown that in rat neonatal hippocampus subjected to hypoxic ischemia the overall amount of complex gangliosides (GM1, GD1a, GD1b, GT1b) decreases compared to controls (Ramirez et al., 2003). In the middle cerebral artery occlusion (MCAO) mouse model of stroke, a similar decrease in the levels of complex gangliosides, compared to the contralateral unaffected hemisphere, occurred only 14 days post-MCAO, and was preceded by a temporary increase of their levels at earlier timepoints (24–72 h). In addition, a specific increase of d20:1 GM1 species was found at the border of the infarcted area. GM2 and GM3 were also found to be increased at the border of the infarcted tissue 3–7 days post MCAO, at a time that is usually associated with inflammation and secondary neuronal death. The authors of this study proposed that the shift from complex to simple gangliosides might be due to the catabolism of complex gangliosides with accumulation of deriving GM2 and GM3 in the lysosomes (Whitehead et al., 2011). In support of this hypothesis, administration of chloroquine in rats, prior to and after stroke, to block ganglioside catabolism, prevented the shift from complex to simple gangliosides as well as motor deficits, decreased inflammation and increased cell survival at the injury site (Caughlin et al., 2019). These data suggest that the depletion of complex gangliosides due to their catabolism might contribute to tissue damage in stroke models and that increasing endogenous ganglioside synthesis might have beneficial effects. In line with this hypothesis, treatment of rats subjected to ischemic stroke with L-threo-1-phenyl-2-decanoylamino-3-morpholino-1-propanol (L-PDMP), an activator of glucosylceramide synthase and ganglioside synthesis, was shown to decrease behavioral deficits (Hisaki et al., 2008).

Several lines of investigation show that administration of GM1 has beneficial effects in stroke and ischemia models. In rats with MCAO, where activation of autophagy exacerbates brain damage and cell death (Li et al., 2018), administration of GM1 resulted in the inhibition of autophagy and in neuroprotection (Li et al., 2016). In rat models of cerebral ischemia-reperfusion injury, GM1 administration decreased brain levels of excitotoxic amino acids as well as markers of oxidative stress (Zhang et al., 2015), downregulated NMDA receptors involved in excitotoxicity, and reduced infarct size (Liu et al., 2005). In a diabetic rat model of stroke, where brain damage is caused by upregulation of ER stress (Srinivasan and Sharma, 2011) and phosphorylation of ERK1/2 (Li et al., 2001), GM1 administration inhibited both and decreased brain damage and neurological deficits (Zhang et al., 2010; Su et al., 2017). GM1 also decreased cerebral edema and cognitive and motor impairment in a rat model of high altitude-induced hypoxia, via activation of the PI3K/AKT-Nrf2 pathway (Gong et al., 2018). Finally, it has been suggested that an additional mechanism of neuroprotection by GM1 in stroke models could be through the restoration/protection of paranodal regions in myelinated axons and prevention of myelin sheath rarefaction (Zhang et al., 2011).

### Multiple Sclerosis

Multiple sclerosis (MS) is the most common progressive neurological disorder in young adults (Browne et al., 2014). It is characterized by a chronic autoimmune reaction against myelin sheaths, resulting in lesions in the brain and spinal cord and neurological dysfunction (Keggan and Noseworthy, 2002).

Measurements of ganglioside levels in the CSF and blood of MS patients are inconsistent (Miyatani et al., 1990; Trbojevic-Cepe et al., 1991) and show dynamic changes with disease state and progression (Lebrun and Cherayil, 1976; Trbojevic-Cepe et al., 1991; Zaprianova et al., 2001), the pathophysiological relevance of which is difficult to determine as they might be related to inflammation as well as demyelination/remyelination processes.

Several studies have tested the effects of exogenously added gangliosides in animal models of MS and demyelinating diseases. In experimental allergic encephalomyelitis (EAE) models, administration of a mix of brain gangliosides subdued the immune response of pathogenic Th1 cells and suppressed the development of clinical symptoms (Shimada et al., 1994; Inoue et al., 1996; Sekiguchi et al., 2001; Castro et al., 2004). These observations contradict early reports describing the development of an experimental autoimmune MS−like disease following administration of gangliosides in healthy rabbits (Konat et al., 1982); and the increase in the production of Th1 with a concomitant decrease of Th2 cytokines in phytohemagglutinin-stimulated human T cells incubated with GD1b, GT1b, and GQ1b *in vitro* (Kanda and Watanabe, 2001). The reason for these discrepancies is unclear, but we speculate that it might relate to the different mixes of gangliosides used, their purity and/or the presence of potential contaminants and endotoxins. Thus, whether specific gangliosides might provide protection in MS models remains unclear.

Exogenous administration of GM4 or GD1a induces oligodendrocytes proliferation and differentiation (Qin et al., 2017; Kieser et al., 2018), a process that is important for remyelination and is impaired in MS (Franklin and Ffrench-Constant, 2008). One of the reasons for limited remyelination in MS is the persistent presence of aggregates of the extracellular matrix protein fibronectin in MS lesions, resulting in inhibition of oligodendrocyte progenitor cell (OPC) maturation and differentiation (Stoffels et al., 2013). The exogenous addition of GD1a was shown to overcome these inhibitory effects, to stimulate OPC proliferation and maturation, and to promote remyelination *in vitro* and *in vivo* in the cuprizone model of demyelination (Qin et al., 2017). These findings suggest that GD1a could have a potential role as a remyelinating agent.

### Depression and Anxiety

Mood disorders such as depression and anxiety are often present in all the neurodegenerative conditions discussed above, and can precede the onset of other symptoms by many years (Galts et al., 2019). There is a wealth of evidence implicating monoamines such as dopamine, norepinephrine and serotonin (5-HT) and their receptors in mood regulation and mood disorders (Jesulola et al., 2018). The ability of gangliosides to serve as co-receptors for these neurotransmitters and to facilitate their access to membrane-embedded ligand-binding sites (Price et al., 1979; Matinyan et al., 1989; Postila et al., 2016; Juhola et al., 2018) suggests they could play a role in mood disorders. This hypothesis is further supported by evidence that pharmacological inhibition of glycosphingolipid synthesis results in decreased ligand binding to 5-HT1A and 5-HT7a receptors, as well as decreased 5-HT1A receptor levels at the plasma membrane (Singh et al., 2012). On the other hand, administration of exogenous GQ1b, and to a lesser extent GM1, were shown to increase the affinity of 5-HT1 receptors for serotonin and coupling of the receptor-adenylate cyclase complex (Berry-Kravis and Dawson, 1985). Molecular dynamics simulations suggests that the glycan moiety of GM1 interacts with a sphingolipid-binding domain (SBD) located in the extracellular loop of the 5-HT1a receptor and drives a conformational change that might favor ligand binding (Chattopadhyay et al., 2012; Wheatley et al., 2012). While it is not known whether gangliosides can have similar effects on dopamine receptors, incubation of rat synaptosomes with GM1 was shown to increase the affinity of the neuronal dopamine transporter (DAT) for dopamine and to decrease its V_*max*_, reflecting a slight reduction of the number of functional uptake sites (Barrier et al., 2003). Altogether, this evidence suggests that membrane ganglioside levels could modulate serotonergic and dopaminergic neurotransmission.

A few studies have shown anti-depressant and anxiolytic effects of exogenously administered gangliosides or GM1 in animal models of ethanol consumption (Gilmore et al., 1991), ethanol withdrawal (Wallis et al., 1995), as well as chronic social defeat stress (Jiang et al., 2016). GM1 administration also reduced depression-like and anxiety-like symptoms in models of HD and in aging wild-type animals (Alpaugh et al., 2017). These effects were accompanied by an increase in the cortical brain levels of serotonin and a decrease in the levels of the product of serotonin catabolism, 5-HIIA, indicating decreased serotonin turnover in mice treated with GM1 (Alpaugh et al., 2017). Thus, the emerging picture is that GM1 might be a modulator of serotonergic transmission.

## Clinical Applications of GM1

Of all major brain gangliosides, GM1 is the only one that has been extensively investigated in clinical studies. This is likely a reflection of the overwhelming focus on this ganglioside, compared to others, in early preclinical work on neuroprotection (Mocchetti, 2005; Magistretti et al., 2019). GM1 has been tested in several human trials in PD, stroke, spinal cord injury and AD, which have demonstrated a good safety profile for this ganglioside. An extensive review of these studies was published previously (Magistretti et al., 2019) and here we will just summarize the most salient outcomes and conclusions.

In PD patients, GM1 administration by subcutaneous injection was well tolerated for up to 5 years (Schneider et al., 2010). A randomized, double-blind, placebo-controlled study in 45 patients found significant improvements in the Unified Parkinson’s Disease Rating Scale (UPDRS) motor score after 16 weeks of treatment (Schneider et al., 1998). Patients from this study were subsequently enrolled in a 5-year open-label study and maintained improvements in UPDRS motor score compared to the placebo group – an observation that would be uncharacteristic of a purely symptomatic treatment (Schneider et al., 2010). In a subsequent double-blind, placebo-controlled, delayed-start study, patients who received GM1 from the beginning of the trial showed significant decrease (improvement) in their UPDRS motor score (Schneider et al., 2013). Delayed-start patients, whose symptoms worsened prior to the start of GM1 treatment, improved over the duration of GM1 treatment started at 24 weeks, although did not reach the benefits obtained in patients that had started GM1 earlier (Schneider et al., 2013). This study also showed that continued GM1 use (up to 120 weeks) slowed down the rate of symptoms progression, suggesting that GM1 might have disease-modifying effects in PD.

The largest trials with GM1 were conducted in stroke patients (>2000 patients in total), with GM1 administered intravenously or intramuscularly (Magistretti et al., 2019). Overall, GM1 was safe but provided only modest benefits, if any, in all of the largest trials (Argentino et al., 1989; Lenzi et al., 1989; Alter et al., 1994).

In a large double-blind, randomized, placebo-controlled trial in patients with acute traumatic spinal cord injury, GM1 given intravenously resulted in a significantly faster recovery compared to placebo, although the final extent of recovery was not different between the two groups (Geisler et al., 2001).

Two clinical studies tested the efficacy of ganglioside GM1 for the treatment of AD patients. In a double-blind placebo-controlled study, peripheral administration of GM1 by intramuscular injection did not improve cognitive symptoms (Flicker et al., 1994). In a second study, intraventricular infusion of ganglioside GM1 for up to 1 year in five AD patients improved several cognitive outcomes, and patients reported improved well-being (Svennerholm et al., 2002). However, the very small sample size and uncontrolled design of this study precludes any definitive conclusions on the efficacy of GM1 as a treatment for AD.

Recently, fluorinated [^18^F]F-GM1 was used to assess brain penetration of the ganglioside after intravenous administration in cynomolgus monkeys, by positron emission tomography (PET). This study demonstrated that only 0.4% of the injected dose entered the brain, without any increase over a period of 4 h (Revunov et al., 2020). Thus, poor brain penetration of peripherally injected GM1 likely explains the negative or modest results obtained in past clinical trials.

## Additional Considerations and Conclusion

Novel functions, interactions and pharmacological properties of gangliosides continue to emerge and provide an ever evolving picture of these molecules that were described as a “factotum of nature” (Ledeen and Wu, 2015). Evidence from human and mouse studies strongly suggests that a decrease in endogenous gangliosides plays a significant role in the pathogenesis of PD and HD, although it remains unknown why this pathway becomes affected and whether CSF levels of gangliosides might be used as biomarkers of disease onset and/or progression. For the other neurological conditions discussed in this review, the evidence for a significant contributing role of gangliosides to pathogenesis is less compelling. The case of AD deserves special attention, as GM1 has traditionally been implicated in the amyloidogenic process as a seed for Aβ (Matsuzaki, 2020), yet *in vivo* evidence suggests that the relationship between the ganglioside and AD pathology might be more complex than anticipated, with GM1 and perhaps other a-series gangliosides even playing a protective role (Bernardo et al., 2009; Oikawa et al., 2009; Yang et al., 2013; Dai et al., 2017). Clarifying the current controversy might guide future therapeutic efforts.

The pharmacological use of exogenous GM1 has been shown to have remarkable beneficial and neuroprotective effects across models of neuronal injury and neurodegeneration, regardless of endogenous ganglioside levels, suggesting that ganglioside-based therapies might provide benefits beyond restoring normal levels of GM1. Early efforts to elucidate the neuroprotective action of gangliosides were mainly focused on their neurotrophin-like effects (Mocchetti, 2005), including the ability of GM1 to stimulate Trk receptors and downstream protective pathways, and to induce neurotrophin synthesis and/or release (Fadda et al., 1993; Ferrari and Greene, 1996; Rabin et al., 2002; Lim et al., 2011; Chiricozzi et al., 2017). However, studies in ganglioside knock-out mice and in models of CNS diseases have taught us that a variety of other different mechanisms might mediate the beneficial effects of exogenously administered GM1, besides and independently from canonical neurotrophic effects. The ability of gangliosides to interact with amyloidogenic proteins such as Aβ, α-synuclein and mHTT, and to modulate how these proteins fold and misfold might be particularly important from a mechanistic and therapeutic perspective, and deserves close attention. Potent anti-inflammatory effects of GM1 on microglia have also been recently described (Galleguillos et al., 2020). As aberrant and chronic microglia activation and neuroinflammation are important features of many neurodegenerative disorders (Perry et al., 2010), this property of GM1 could further contribute to ameliorate neurodegenerative conditions.

Overall, the demonstrated safety profile of GM1 and the compelling evidence of its disease-modifying effects in HD (Di Pardo et al., 2012; Alpaugh et al., 2017) and in PD (Schneider et al., 2019) warrant further clinical investigation in patients. The development of strategies to overcome issues with drug delivery across the blood-brain barrier (BBB) (Revunov et al., 2020) will be crucial to this endeavor, even for the treatment of neurodegenerative/trauma-related conditions where the BBB might be partially compromised. Recent studies suggested that the pentasaccharide component of the GM1 molecule, which can reproduce the ganglioside effects on TrkA receptor activation and signaling (Chiricozzi et al., 2017; Di Biase et al., 2020a), can cross an *in vitro* model of BBB through a paracellular route (Di Biase et al., 2020b). However, whether the pentasaccharide can cross the BBB in more stringent conditions and *in vivo*, where the paracellular transport is very limited (Sweeney et al., 2019) remains to be determined. Interestingly, GM1 pentasaccharide was shown to provide beneficial effects similar to those exerted by GM1 in the B4galnt1^+/–^ mouse model of parkinsonism (Chiricozzi et al., 2019). Caution should be exerted in extending these findings to other models. In the absence of a ceramide tail that would anchor the molecule to membranes, the pentasaccharide is unlikely to reproduce many crucial GM1 functions. On the other hand, GM1 analogs that retain membrane anchoring properties might mimic the pleiotropic effects of GM1 more closely.

## Author Contributions

SS conceived the review’s plan and organization, wrote the review, and prepared the figures. JM contributed to the writing and to the preparation of the table. DG contributed to the writing and to the preparation of the table, and prepared the figures. NS and VK contributed to the writing and to the preparation of the table. All authors contributed to the article and approved the submitted version.

## Conflict of Interest

SS and the University of Alberta hold a patent for the use of GM1 in HD. The remaining authors declare that the research was conducted in the absence of any commercial or financial relationships that could be construed as a potential conflict of interest.
